# Integrated disease management: arboviral infections and waterborne diarrhoea

**DOI:** 10.2471/BLT.20.269985

**Published:** 2021-04-29

**Authors:** Hans J Overgaard, Nsa Dada, Audrey Lenhart, Thor Axel B Stenström, Neal Alexander

**Affiliations:** a Faculty of Science and Technology, Drøbakveien 31, Norwegian University of Life Sciences, NO – 1432 Ås, Norway.; b Center for Global Health, United States Centers for Disease Control and Prevention, Atlanta, United States of America.; c Institute for Water and Wastewater Technology, Durban University of Technology, Durban, South Africa.; d MRC Tropical Epidemiology Group, London School of Hygiene and Tropical Medicine, London, England.

## Abstract

Water-related diseases such as diarrhoeal diseases from viral, bacterial and parasitic organisms and *Aedes*-borne arboviral diseases are major global health problems. We believe that these two disease groups share common risk factors, namely inadequate household water management, poor sanitation and solid waste management. Where water provision is inadequate, water storage is essential. *Aedes* mosquitoes commonly breed in household water storage containers, which can hold water contaminated with enteric disease-causing organisms. Microbiological contamination of water between source and point-of-use is a major cause of reduced drinking-water quality. Inadequate sanitation and solid waste management increase not only risk of water contamination, but also the availability of mosquito larval habitats. In this article we discuss integrated interventions that interrupt mosquito breeding while also providing sanitary environments and clean water. Specific interventions include improving storage container design, placement and maintenance and scaling up access to piped water. Vector control can be integrated into sanitation projects that target sewers and drains to avoid accumulation of stagnant water. Better management of garbage and solid waste can reduce the availability of mosquito habitats while improving human living conditions. Our proposed integration of disease interventions is consistent with strategies promoted in several global health frameworks, such as the sustainable development goals, the global vector control response, behavioural change, and water, sanitation and hygiene initiatives. Future research should address how interventions targeting water, sanitation, hygiene and community waste disposal also benefit *Aedes*-borne disease control. The projected effects of climate change mean that integrated management and control strategies will become increasingly important.

## Résumé

Diarrhées provoquées par la présence d'organismes viraux, bactériens et parasites, arboviroses véhiculées par les moustiques *Aedes*: les maladies liées à l'eau constituent un problème de santé majeur dans le monde. Nous pensons que ces deux groupes de maladies partagent les mêmes facteurs de risque, à savoir une mauvaise gestion de l'eau au sein du foyer ainsi qu'un manque d'assainissement et de traitement des déchets solides. Dans les endroits où l'approvisionnement en eau est insuffisant, les conditions de conservation sont essentielles. Les moustiques *Aedes* se reproduisent fréquemment dans les réservoirs d'eau à domicile, qui peuvent dès lors contenir de l'eau contaminée par des organismes responsables d'infections entériques. La contamination microbiologique de l'eau, entre la source et le moment où elle est consommée, représente l'une des causes principales d'altération de la qualité de l'eau potable. Le manque d'assainissement et de traitement des déchets solides fait augmenter le risque de contamination de l'eau, mais aussi le nombre de biotopes disponibles pour les larves de moustique. Dans cet article, nous parlons des interventions intégrées qui permettent d'interrompre la reproduction des moustiques tout en créant des environnements sanitaires adaptés et de l'eau propre. Ces interventions spécifiques prévoient notamment une optimisation de la conception, du placement et de l'entretien des réservoirs, ainsi qu'un meilleur accès à l'eau courante. La lutte contre les vecteurs peut être incorporée dans des projets d'assainissement qui ciblent les égouts et canalisations, afin d'éviter toute accumulation d'eau stagnante. Une meilleure gestion des ordures ménagères et des déchets solides peut réduire le nombre de biotopes disponibles pour les moustiques, mais aussi améliorer les conditions de vie de la population. Nous proposons une gestion intégrée des maladies cohérente avec les stratégies mises en avant dans plusieurs cadres de santé mondiaux tels que les objectifs de développement durable, l'action mondiale pour lutter contre les vecteurs, le changement de comportement ainsi que les initiatives relatives à l'approvisionnement en eau, l'assainissement et l'hygiène. Les futures recherches devraient étudier la façon dont les interventions dédiées à l'eau, à l'assainissement, à l'hygiène et à l'élimination des déchets au sein des communautés contribuent également à la lutte contre les maladies véhiculées par les moustiques *Aedes*. Compte tenu des effets attendus du changement climatique, les stratégies de lutte et de gestion intégrée vont gagner en importance.

## Resumen

Las enfermedades relacionadas con el agua, como las enfermedades diarreicas por organismos víricos, bacterianos y parasitarios, y las enfermedades arbovirales transmitidas por el *Aedes*, son importantes problemas sanitarios a nivel mundial. Creemos que estos dos grupos de enfermedades comparten factores de riesgo comunes, es decir, una gestión inadecuada del agua en los hogares, un saneamiento deficiente y la gestión de los residuos sólidos. Cuando el suministro de agua es inadecuado, el almacenamiento de agua es esencial. Los mosquitos *Aedes *suelen criar en los recipientes de almacenamiento de agua de los hogares, que pueden contener agua contaminada con organismos causantes de enfermedades entéricas. La contaminación microbiológica del agua entre la fuente y el punto de uso es una de las principales causas de la reducción de la calidad del agua potable. Un saneamiento y una gestión de residuos sólidos inadecuados no solo aumentan el riesgo de contaminación del agua, sino también la disponibilidad de hábitats para las larvas de mosquitos. En este artículo se analizan las intervenciones integradas que interrumpen la cría de mosquitos al tiempo que proporcionan entornos sanitarios y agua limpia. Las intervenciones específicas incluyen la mejora del diseño, la colocación y el mantenimiento de los contenedores de almacenamiento y la ampliación del acceso al agua corriente. El control de los vectores puede integrarse en proyectos de saneamiento dirigidos a las alcantarillas y los desagües para evitar la acumulación de agua estancada. Una mejor gestión de la basura y los residuos sólidos puede reducir la disponibilidad de hábitats para los mosquitos y mejorar las condiciones de vida de las personas. Nuestra propuesta de integración de las intervenciones contra la enfermedad es coherente con las estrategias promovidas en varios marcos sanitarios mundiales, como los objetivos de desarrollo sostenible, la respuesta mundial de control de vectores, el cambio de comportamiento y las iniciativas de agua, saneamiento e higiene. La investigación futura debería abordar cómo las intervenciones dirigidas al agua, el saneamiento, la higiene y la eliminación de residuos de la comunidad también benefician al control de las enfermedades transmitidas por el *Aedes*. Los efectos previstos del cambio climático significan que las estrategias de gestión y control integrados serán cada vez más importantes.

## ملخص

تعتبر الأمراض المرتبطة بالمياه مثل أمراض الإسهال الناتجة عن الكائنات الفيروسية والبكتيرية والطفيلية، والأمراض الفيروسية المنقولة عن طريق بعوض الزاعجة، من المشكلات الصحية العالمية الرئيسية. نحن نعتقد أن هاتين المجموعتين من الأمراض تشتركان في عوامل خطر مشتركة، وهي الإدارة غير المناسبة للمياه المنزلية، وسوء الصرف الصحي وإدارة النفايات الصلبة. عندما يكون توفير المياه غير كافٍ، يكون تخزين المياه ضروريًا. يتكاثر بعوض الزاعجة عادة في حاويات تخزين المياه المنزلية، والتي يمكن أن تحتوي على مياه ملوثة بالكائنات المسببة للأمراض المعوية. يعد التلوث الميكروبيولوجي للمياه بين المصدر ونقطة الاستخدام سببًا رئيسيًا لانخفاض جودة مياه الشرب. إن الصرف الصحي غير الملائم وسوء إدارة النفايات الصلبة لا يزيد فقط من خطر تلوث المياه، ولكن أيضًا من توافر موائل يرقات البعوض. نحن نناقش في هذه المقالة التدخلات المتكاملة التي توقف تكاثر البعوض مع توفير البيئات الصحية والمياه النظيفة. تشمل التدخلات المحددة تحسين تصميم حاويات التخزين، ووضعها وصيانتها وتوسيع نطاق الحصول على المياه المنقولة بالأنابيب. يمكن دمج مكافحة الحشرات الناقلة للأمراض في مشروعات الصرف الصحي التي تستهدف المجاري والمصارف لتجنب تراكم المياه الراكدة. يمكن أن تؤدي الإدارة الأفضل للقمامة والنفايات الصلبة إلى تقليل توافر موائل البعوض، جنباً إلى جنب مع تحسين الظروف المعيشية للإنسان. يتوافق التكامل الذي نقترحه لتدخلات الأمراض مع الاستراتيجيات التي تم الترويج لها في العديد من أطر العمل الصحية العالمية، مثل أهداف التنمية المستدامة، والاستجابة العالمية لمكافحة الحشرات الناقلة للأمراض، وتغيير السلوك، ومبادرات المياه والصرف الصحي والنظافة. يجب أن تتناول الأبحاث المستقبلية كيف يمكن للتدخلات التي تستهدف المياه والصرف الصحي والنظافة والتخلص من النفايات المجتمعية، أن تستفيد أيضًا من مكافحة الأمراض التي تنقلها بعوض الزاعجة. إن الآثار المتوقعة لتغير المناخ تعني أن استراتيجيات الإدارة والتحكم المتكاملة ستصبح ذات أهمية متزايدة.

## 摘要

水相关疾病,如由病毒、细菌和寄生虫引起的腹泻疾病以及*伊蚊*传播的虫媒病毒疾病,是主要的全球卫生问题。我们认为,这两个疾病组有共同的风险因素,即家庭用水管理不足、卫生条件差和固体废物管理不善。在供水不足的地方,水的储存是必不可少的。*伊蚊*通常在家庭储水容器中繁殖,这些容器中可能含有被肠道致病微生物污染的水。水源和用水点之间的微生物污染是饮用水质量下降的一个主要原因。卫生设施和固体废物管理不足不仅增加了水污染的风险,而且还成了蚊子幼虫繁殖的温床。在本文中,我们讨论了在提供卫生环境和清洁水源的同时,中断蚊子繁殖的综合干预措施。具体的干预措施包括,改进存储容器的设计、放置和维护,以及扩大管道供水的使用。可将病媒控制纳入以下水道和排水沟为目标的卫生项目,以避免积水。更好地管理垃圾和固体废物可以减少蚊子栖息地,同时改善人类的生活条件。我们提出的疾病干预综合措施符合可持续发展目标、全球病媒控制对策、行为改变以及水、环境卫生和个人卫生倡议等若干全球卫生框架所倡导的战略。未来的研究应解决以水、环境卫生、个人卫生和社区废物处理为目标的干预措施如何同时助益*伊蚊*传播疾病的控制。气候变化的预期影响意味着综合管理和控制战略将变得越来越重要。

## Резюме

Болезни, передаваемые через воду, такие как диарейные заболевания, вызванные вирусными, бактериальными и паразитарными организмами, а также арбовирусные заболевания, передаваемые через инфицированных комаров вида *Aedes*, являются серьезными глобальными проблемами здравоохранения. Авторы считают, что эти две группы заболеваний имеют общие факторы риска, а именно: неадекватное управление водными ресурсами в домашних хозяйствах, плохую санитарию и удаление твердых отходов. При недостаточном водоснабжении крайне важно обеспечить запасы воды. Комары вида *Aedes* обычно размножаются в бытовых резервуарах для хранения воды, в которых может содержаться вода, загрязненная кишечными болезнетворными организмами. Микробиологическое загрязнение воды между источником и местом использования является основной причиной снижения качества питьевой воды. Недостаточная санитария и неэффективное обращение с твердыми отходами не только повышают риск загрязнения воды, но и обеспечивают наличие мест обитания личинок комаров. В этой статье рассматриваются комплексные меры, препятствующие размножению комаров и обеспечивающие санитарные условия и чистую воду. Конкретные меры включают совершенствование конструкции резервуаров для хранения, их размещения и технического обслуживания, а также расширение доступа к водопроводной воде. Борьба с переносчиками инфекции может быть включена в проекты санитарной обработки, нацеленные на канализацию и стоки во избежание накопления застойной воды. Более эффективное обращение с мусором и твердыми отходами может снизить доступность мест обитания для комаров, улучшив при этом условия жизни людей. Предлагаемое авторами комплексное осуществление мер по борьбе с болезнями согласуется со стратегиями, продвигаемыми в нескольких глобальных планах здравоохранения, таких как цели в области устойчивого развития, глобальные меры борьбы с переносчиками болезней, изменение поведенческих моделей и инициатив в области водоснабжения, санитарии и гигиены. В будущих исследованиях следует рассмотреть, как меры, направленные на водоснабжение, санитарию, гигиену и удаление бытовых отходов, также могут помочь в борьбе с болезнями, переносимыми комарами *Aedes*. Прогнозируемые последствия изменения климата означают, что комплексные стратегии управления и контроля будут приобретать все большее значение.

## Introduction

Water-related diseases such as diarrhoeal diseases from viral, bacterial and parasitic organisms and *Aedes*-borne arboviral diseases are major global health problems (Box 1; Table 1). The effects of water on disease are determined by multiple factors including the water source, pathogen abundance and diversity, and human water management practices. For waterborne diarrhoeal diseases, these determinants relate to faecal contamination at the water source, in transit and during storage, while for diseases borne by *Aedes* spp. mosquitoes, such as dengue fever, Zika virus disease and chikungunya, the determinants relate to water storage functioning as mosquito larval habitats.

Box 1 **Risk factors and burden of dengue and diarrhoeal diseases**

Water-related diseases may be classified into waterborne, such as diarrhoeal diseases; water-based, such as schistosomiasis; and water-related vector-borne, such as dengue.^1^

Dengue, Zika virus and chikungunya arboviral diseases are major global causes of morbidity and mortality sharing the same water-related risk factors and vector species (Table 1).^2^ The main vector, *Aedes aegypti*, commonly breeds in clean water in household water containers in urban areas and is highly anthropophagic, endophilic and diurnal. The larval habitats of *Ae. aegypti* proliferate in areas where water supply is unreliable or where conventional water storage habits persist.^3^ Solid waste production (garbage) and inadequate disposal also result in the accumulation of larval habitats.^4^ A lack of clear evidence of the effectiveness of existing vector control methods indicates that innovative vector control strategies, socioecological approaches and controlled experimental studies are needed.^5^ Determining the disease burden from dengue is impeded by diagnostic difficulties, poor surveillance, low fatality rates and a general lack of intersectoral coordination.^6^^,^^7^

Diarrhoeal diseases are responsible for some of the highest mortality rates worldwide, particularly in young children and people who are malnourished or have impaired immunity (Table 1).^8^ In locations where water provision is inadequate, communities must rely on water harvesting, transport and storage in or near houses for domestic purposes. Microbial contamination between source and point-of-use is often an important cause of reduced quality of household drinking water.^9^ The fraction of diarrhoeal diseases attributable to inadequate water, sanitation and hygiene practices in low- and middle-income countries is about 60% (an estimated 829 000 deaths out of 1.4 million total deaths in 2016).^10^

**Table 1:** Characteristics of dengue and diarrhoeal diseases

Factor	Dengue	Diarrhoeal diseases
Definition and symptoms	A mosquito-borne viral disease which causes influenza-like illness that occasionally develops potentially lethal complications. Typical symptoms include sudden onset of fever, headache, muscle, joint and bone pain	Viral, bacterial and parasitic diseases characterized by the passage of three or more loose or liquid stools per day, or more frequent passage than is normal for the individual^11^
Clinical types	Dengue with or without warning signs. Severe dengue (dengue haemorrhagic fever, dengue shock syndrome)	Acute watery diarrhoea: lasts several hours or days, and includes cholera. Acute bloody diarrhoea, also called dysentery. Persistent diarrhoea: lasts 14 days or longer
Biological agents	Four serotypes of a single-stranded RNA flavivirus: DENV1, DENV2, DENV3, DENV4	Rotavirus, *Shigella* spp. and *Salmonella* spp. are the leading causes of infection leading to death from diarrhoea^8^
Routes of transmission	By mosquito bites. Main mosquito vectors: *Aedes aegypti* (more common in tropical areas) and *Ae. albopictus* (more common in temperate areas). Sexual human-to-human transmission has been reported^12^	By consumption of food or water contaminated with human or animal faecal matter and other causative pathogens. By person-to-person transmission, aggravated by poor personal hygiene and sanitation^8^
Morbidity	Estimated 390 million cases annually. 2.5–3.6 billion people living in risk areas globally^2^	Estimated > 957 million episodes per year.^8^ Occurring globally
Mortality	Estimated average 9200 annual deaths (maximum 11 300) during 1990–2010^13^	Estimated 1.3–1.4 million deaths annually, of which about 499 000 (36%) are in children younger than 5 years^8^^,^^10^
Disability-adjusted life year (DALY)	Dengue was responsible for an estimated 1.14 million (95% uncertainty interval: 0.73–1.98 million) DALYs in 2013^13^	Diarrhoeal diseases are responsible for an estimated 71.6 million DALYs per year (95% uncertainty interval: 66.4–77.2).^8^ The disease burden attributable to water, sanitation and hygiene amounts to 49.8 million global DALYs^10^
Distribution of global burden	Regional distribution of apparent and inapparent infections of the total 390 million dengue infections: Asia, 69.5% (271 million); Americas, 13.8% (53.8 million); Africa, 16.4% (64.1 million)^2^	Regional distribution of episodes out of the total 2.4 billion diarrhoea episodes in all ages: sub-Saharan Africa, 33.5% (801 million); South Asia, 37.6% (899 million); South-East Asia and Oceania, 12.9% (308 million); North Africa and Middle East, 7.1% (170 million); Latin America and Caribbean, 7.2% (172 million); central Europe, eastern Europe, central Asia, 1.3% (31 million); high-income countries, 0.5% (11 million)^8^
Setting	Generally household-centred, mainly in urban, but also in rural areas. Public areas, such as schools, underground drains, industrial and abandoned sites also contribute to mosquito breeding	Generally household-centred, in both urban and rural areas. Public water services may also contribute to water contamination
Risk factors	Interactions between socioeconomic, environmental and behavioural factors such as inadequate water supply, poor water storage and inadequate sanitation conditions. Rapid unplanned and unregulated urbanization, globalization and international travel are global risk factors^14^	Contaminated food and water. Interactions between socioeconomic, environmental and behavioural factors such as inadequate water supply, poor water storage and inadequate sanitation conditions
Treatment and prevention or control	No specific treatment or effective drugs are available. Several vaccine candidates are under various stages of development.^15^ Mosquito control, by chemical, biological or physical means, remains critical for sustained dengue control^5^	Drugs and vaccines are available for some causative pathogens. Access to safe drinking water, improved sanitation, good personal and food hygiene, together with health education, can reduce transmission^8^
Projected effects of climate change	Both future contraction and expansion of areas at risk for dengue have been projected.^16^ Most predictions expect negative impacts of climate change on dengue. An increase in the ability of mosquitoes to transmit dengue and more people being exposed to climates suitable for dengue create greater potential for epidemics of dengue.^17^ Causal pathways are complex because of the intermediate direct and indirect effects on the vector, virus and transmission, further complicated by human behaviour and immunity. Temperature effects are potentially more predictable than independent effects of rainfall and humidity. Increases in temperature will generally increase vector development, survival, density and vector competence, and consequently virus circulation and transmission^17^	Most predictions expect an increase in diarrhoeal diseases (except viral diarrhoea) due to climate change.^18^ Increases in temperature, heavy rainfall, drought and flooding are factors associated with climate change which can result in surface runoff, contamination of drinking-water resources, overwhelmed sanitation and water provision infrastructures at private and public levels, as well as population displacement^18^

RNA: ribonucleic acid.

Storage of water for human consumption and water management practices in both the domestic and public domains are shared risk factors for the transmission of the dengue virus (representative of *Aedes*-borne arboviral diseases in this article) and diarrhoea (here representing a multitude of gastrointestinal diseases). Other potential shared risk factors are inadequate sanitation and waste disposal.^4^^,^^19^ Targeting such risk factors allows for integrated disease control and risk management. Co-occurrence and coinfection of both diarrhoeal diseases and dengue may explain the shared epidemiology of the diseases and can guide the design of integrated management strategies. In this article, we propose options for integrated interventions and how they fit into established health and development frameworks. We discuss considerations around sustainability of interventions and identify priorities for future research.

## Common factors 

Knowing the geographical co-occurrence of diseases is important for allocating scarce resources. Globally, the burden of diarrhoeal diseases is highest in Africa,^8^ whereas dengue is highest in Asia.^13^ However, recent research on dengue in Africa has shown that it is more prevalent than previously thought.^20^ Some regions, notably the Caribbean (such as Haiti and Suriname) and Asia (such as India, Indonesia, Lao People's Democratic Republic and the Philippines), have a high incidence of both dengue and diarrhoeal diseases.^8^^,^^13^ Spatial overlap may be more evident at smaller scales, because more detailed spatial and temporal variation in disease prevalence is not fully reflected in national-level statistics. Diarrhoeal diseases are more widespread and their burden is orders of magnitude higher than dengue. The more geographically constrained distribution of dengue could therefore provide a starting point for identifying locations suitable for integrating management strategies within areas where the two diseases overlap.

**Fig. 1:**
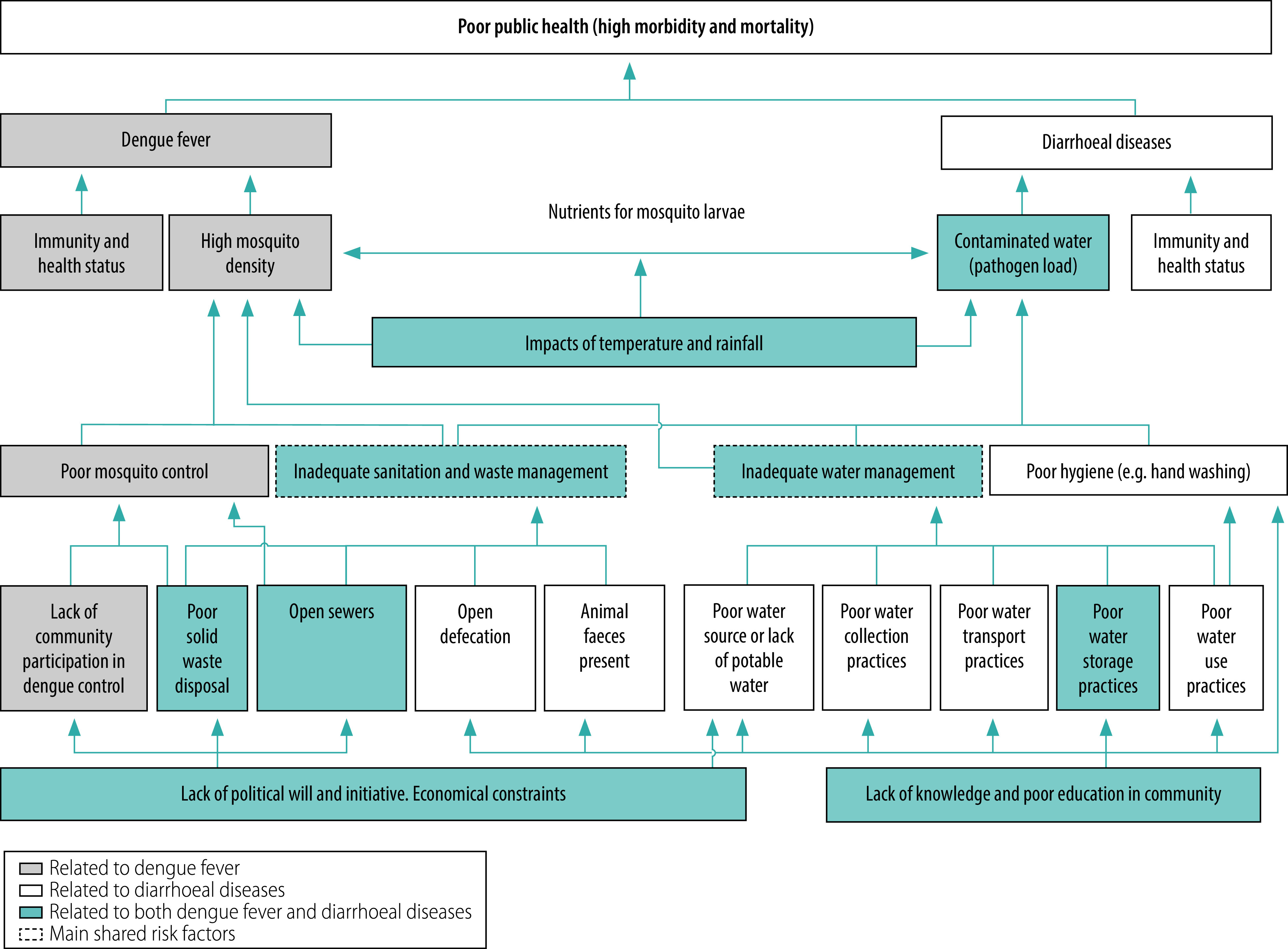
Problem analysis of the determinants of dengue and diarrhoeal diseases

Note: The figure shows a problem tree done using a logical framework approach indicating cause and effect relationships between dengue (representing *Aedes*-borne arboviral diseases) and waterborne diarrhoeal diseases.

Understanding the shared risk factors between dengue and diarrhoeal diseases can help identify suitable integrated management and control strategies. We conducted a problem analysis as part of a logical framework approach showing cause and effect relationships between dengue and diarrhoeal diseases (Fig. 1). We identified water storage containers, sanitation and waste disposal as the main shared risk factors. These factors vary by location and time.

Factors related to water management include the source of water and how the water is collected, stored, used and treated, all of which can also affect water quality. Contamination may occur at any of these points, but also through poor sanitation and sewage systems. Poor water management is clearly related to diarrhoeal diseases, but can also contribute to propagation of the vectors of dengue. The nutritional quality of the larval environment affects the size and survival of mosquitoes, which may also impact vector-borne disease transmission.^21^ As such, general contamination or accumulation of organic matter in water can favour larval development. We have previously shown that there are more *Ae. aegypti* pupae in containers that are contaminated with *Escherichia coli* compared with uncontaminated containers.^22^ Inadequate sanitation and solid waste management also affect both diseases as these factors increase the risk of water contamination and the availability of potential mosquito larval habitats.^4^

## International frameworks

As we discuss in the next section, our proposed integrated interventions are closely aligned with the sustainable development goals (SDGs), particularly: strengthening good health and well-being (SDG 3), improving quality education to promote sustainable development (SDG 4), providing clean water and sanitation (SDG 6), making cities and communities safe, resilient and sustainable (SDG 11), reducing the effect of climate change (SDG 13) and supporting global partnerships (SDG 17).

The World Health Organization’s (WHO) *Handbook for integrated vector management* aims to break the traditional top-down, insecticide-based, single-intervention approaches in favour of more evidence-based, integrated and participatory strategies.^7^ Integrated vector management is defined as a rational decision-making process to optimize the use of resources for vector control. Vector control methods should preferably target the vectors of multiple diseases and be implemented through intersectoral collaboration and community participation. Integrated vector management is at the centre of the WHO global vector control response adopted in 2017, which aims to reduce vector-borne disease mortality and incidence in 2030 by at least 75% and 60%, respectively.^6^ This target will be achieved by strengthening intersectoral collaboration, engaging communities, enhancing vector surveillance and scaling up and integrating vector control methods, supported by enhanced capacity and increased research and innovation. The global vector control response recommends comprehensive vector control through integrated action using effective existing and novel vector control approaches. A complementary framework for addressing behavioural change in dengue control is the Communication for Behavioural Impact approach,^23^ which is a planning tool with a mixture of theory and practice. The approach uses communication theory and marketing practices to achieve behaviour change through a broad integration of mobilization, communication, strategic planning and evaluation of specific behaviours.

Waterborne disease control frameworks include interventions related to water, sanitation and hygiene (known as WASH). WHO and others promote household water treatment and safe storage.^24^ Some scientists argue, however, that the evidence for scaling up household water treatment to reduce diarrhoeal diseases is not strong enough and that greater emphasis should be placed on water access and water quantity, rather than water quality.^25^ Nonetheless, household water treatment and safe storage does substantially improve the microbiological quality of water. More than an estimated 60% (risk ratio: 0.39; 95% confidence interval: 0.32–0.48) of diarrhoeal diseases prevalence could be reduced by filtering and safe storage of water.^26^ Water safety plans are international preventive risk management systems developed by WHO to manage, monitor and evaluate drinking-water quality.^27^ The guidelines apply to all kinds of water supply systems from large piped drinking-water supplies to small community and household supply systems. Other researchers have proposed the Integrated Behavioural Model for water, sanitation and hygiene to address behavioural change.^28^ Based on a comprehensive framework, the model includes contextual, psychosocial and technology factors that operate on five different levels: societal (broad organizational, institutional or cultural factors); community (physical and social environment); interpersonal or household (interactions between closely related individuals); individual (sociodemographic factors, such as age and sex); and habits (opportunities and necessities affecting behaviours nested within the individual). The model provides conceptual and practical tools for improving knowledge about and evaluation of factors that influence water, sanitation and hygiene practices to sustain behaviour change in areas with limited infrastructure. The theoretical behavioural frameworks mentioned above are only a small sample of the available evidence-based behavioural theories demonstrated to be suitable and useful in waterborne disease control in general. Finally, these and other related frameworks must be understood in relation to climate resilience,^29^ and community vulnerability and adaptability.^30^

## Integrated disease management

The frameworks we outline provide a foundation for evaluating the suitability of specific interventions for integrated disease control and management. Here we propose the integrated management of diarrhoeal diseases and dengue based on identified shared risk factors (Fig. 1). Integrated management should interrupt mosquito breeding while providing a clean sanitary environment along with clean water. Generally, the household is targeted for integrated disease management, but interventions that focus on non-domestic sites – such as schools, workplaces, hospitals and industrial sites – must also be considered.^6^ In this context, urban spaces need to be classified by their physical accessibility and legal accountability, which may impede access to and failure of assigning responsibility for vector control actions.^31^ Such interventions in society can be helpful in identifying integrated strategies that are suitable for specific locations, employing bottom-up community action as well as government-driven top-down approaches. 

Another consideration is the impact of climate change on these diseases. Increases in temperature and increases or decreases in rainfall, flooding and humidity will likely intensify the epidemic potential and expand areas suitable for transmission of both arboviral and waterborne diarrhoeal diseases.^17^^,^^18^ These changes involve complex causal pathways, including the prevalence of breeding sites; increased survival or prevalence of pathogens and vectors; contamination of drinking-water resources; overwhelmed infrastructures; and population displacements.^17^^,^^18^ The effects of climate change on the seasonality of disease outbreaks may also be important, involving complexities beyond the scope of this article. However, future increases in the occurrence of these diseases would increase the need for integrated management strategies.^30^^,^^32^ To mitigate the effects of climate-related events, early warning systems could be useful for both dengue and diarrhoeal disease surveillance and control.^33^

### Water management

Water management relates to the quantity, quality and accessibility of water, its collection, transport and storage practices, as well as its consumption and treatment patterns. The source of the water can influence its quality, which can affect both its suitability for human consumption and the risk of *Aedes* mosquitoes breeding. A study in southern Lao People's Democratic Republic found that household containers filled with borehole water were almost four times more likely to be infested with *Ae. aegypti* pupae than containers with rain-fed or purchased bottled water.^34^ Containers with borehole water had higher levels of *Escherichia coli* than other containers.^35^ A relationship between *Ae. aegypti* productivity and *E. coli*-contaminated domestic water containers has been found,^22^ although any consequent disease outcomes remain unknown. Water quality is a risk factor for diarrhoeal diseases and potentially also for dengue, since the nutritional quality of larval habitats affect mosquito size and survival, which in turn affect vector capacity.^21^^,^^36^ India has implemented groundwater recharge programmes to manage water crises through a variety of rainwater harvesting structures, such as percolation pits and structures connected to wells (in use or disused). Defective rainwater harvesting structures were found to be key breeding habitats for *Aedes* mosquitoes.^37^ These findings highlight that integrated control interventions targeting the water source should include water quality improvements as well as infrastructure management and repair.

Insufficient supply of water requires the need to store water. Improving the supply and storage of water in domestic and public domains is an obvious target for integrated control of dengue and diarrhoeal diseases. An unreliable drinking-water supply has been associated with higher *Ae. aegypti* indices, such as the presence and proportion of positive containers (container index).^3^ Rural areas with a lack of piped water supply in Viet Nam had a higher risk of dengue than urban areas with an adequate water supply.^38^ However, domestic household water storage is common even in areas with reliable access to piped water, and immature vectors of dengue are still found in such containers.^22^ Simply improving water connections into houses may not necessarily prevent people from storing water.

Interventions targeting water storage containers for integrated control in households should focus on the type, quality and cleanliness of the container. Improving container design is needed, including covers that prevent mosquitoes from breeding and other types of contamination from occurring. Improved design and placement of containers may prevent contamination during flooding and heavy rainfall events that are expected to become more frequent with climate change. If drinking-water containers contribute substantially to the number of mosquitoes produced in an area, then an integrated dengue–diarrhoea control project could have a major impact. The WHO global assessments of household water treatment technologies show that several meet the established microbiological performance criteria in terms of pathogen removal.^24^ Such technologies are based, for example, on various filtration methods using membrane, ceramic or flocculation techniques and disinfection methods using ultraviolet, solar or chemical (chlorine) techniques. However, the effect of these methods on mosquito breeding is not well characterized. Differences in designs of these technologies determine their importance for integrated control. Indeed, inclusion of vector control effects as an additional criterion would enhance the value of these household water treatment assessments. Chlorine has been used to clean containers for vector control but, although effective against bacteria and protozoa, chlorine is less effective against viruses.^24^ Scrubbing the inside walls of washbasins and water storage drums with a mixture of bleach and detergent in households in Honduras showed high mortality rates of *Ae. aegypti* eggs, larvae and pupae.^39^ It is unclear, however, whether maintaining container cleanliness for vector control would also reduce pathogens through chlorine residuals. These findings underscore the importance of appropriate site-specific dosing based on the chlorine demand of the water to be treated. Regular monitoring is also needed to ensure that free residual chlorine concentrations of 0.2–0.5 mg/L are maintained and that these interventions reduce vector breeding.

The physical location of water storage tanks can also provide an opportunity for integrated control. Studies have reported that *Ae. aegypti* pupae are not found in elevated water storage tanks which are located, for example, on a roof or otherwise above the ground, potentially due to heating from direct sun exposure.^40^ Keeping water storage containers out of reach of people or, preferably, installing closed systems that avoid contamination should be considered for integrated dengue and diarrhoeal diseases control. Such interventions will become even more important during climate change when flooding and extreme weather events are likely to become more frequent.

### Sanitation and waste management

A sanitation system includes the capture, storage, transport, treatment and disposal or reuse of human excreta and wastewater. Targeting the sanitation system to reduce water contamination is well known to reduce diarrhoeal diseases, but less is known about its impact on dengue. *Ae. aegypti* can lay eggs in raw sewage, with normal egg hatching and larvae development.^41^ *Aedes* mosquitoes have also been found breeding in subterranean septic tanks and subsurface catch basins, which can contribute substantially to productivity.^42^ As sanitation projects often target sewers and drains, vector control could be incorporated into such projects by avoiding the accumulation of stagnant water and ensuring that vectors are unable to enter physical structures.

Poor solid waste disposal is another potential risk factor for transmission of vector-borne and diarrhoeal diseases. Improperly managed waste such as motor vehicle tyres – implicated in the global spread of *Ae. albopictus*^43^ – provide suitable larval habitats for mosquito vectors as well as increased risk for enteric diseases, particularly for children.^44^ Stockpiles of tyres should be properly stored in ways that avoid water accumulation and reduce mosquito breeding. Deficiencies in public services, such as water supply, waste collection and excreta disposal, can be responsible for high indices of *Ae. aegypti* infestation.^45^ Provision of solid waste management, recycling and repurposing of plastics and tyres, reliable piped water supplies and improved housing design are all key long-term steps towards reducing vector populations and improving environmental health.

## Discussion

Water management, sanitation and waste management are key targets for integrated dengue and diarrhoeal diseases control. Specific water management interventions targeting the water source should include water quality improvements and infrastructure management and repair. Household water treatment and storage interventions should consider improved container design to prevent mosquito breeding and water contamination as well as container cleanliness using disinfection methods, such as chlorine. Awareness of vector control opportunities while planning improvements of sanitation systems, such as physical and organizational structures and facilities, could lead to improved sanitation as well as reduced vector densities. An effective solid waste management system can improve environmental health, human living conditions and the general health of people, while reducing the availability of suitable larval habitats. Integrated interventions in non-residential sites, such as in schools, need careful planning of appropriate sustainable combinations of site-specific, effective, acceptable and sustainable interventions.

The lack of research on integrated dengue and diarrhoeal disease interventions prevents us from drawing conclusions about their benefits. We have found only one trial that assessed an integrated strategy, assessed by a factorial, cluster, randomized controlled design in rural primary schools in Colombia during 2012–2014.^46^ The trial implemented sets of physical and educational interventions targeting dengue and diarrhoeal diseases. Interventions were effective in reducing mosquito larval habitats in schools and in providing clean water; however, students’ absence from school and adult mosquito density in schools were not affected. The study concluded that integrated approaches should not be limited to schools but also implemented simultaneously in communities. Two years after the trial ended the researchers assessed the sustainability of the interventions and institutional adoption in terms of stakeholder empowerment, financial support, participation and leadership, adaptive flexibility and capacity.^47^ These categories were measured using a mixture of knowledge, attitude and practices questionnaires assigned to students and teachers, semi-structured interviews with teachers, as well as observations of the maintenance of the interventions. Both the educational and the physical interventions were considered moderately sustainable, but the institutional and human adoption were considered unsustainable. A lack of adoption of initiatives is not uncommon, where the short-term nature of projects often conflicts with the long-term needs of the community. The researchers further explained the failure of institutional commitment by a lack of integration of the interventions into the activities of schools and municipalities. Integration is a process that requires time and respectful dialogue between project innovators and the educational institutions to generate engagement, enthusiasm and a sense of ownership. From a teaching standpoint, schools should focus on place-based education that promotes learning rooted in local habitats.^48^ Such teaching methods, combined with adapted Communication for Behavioural Impact activities,^23^ can contribute to diffusion of knowledge from schools to communities and can lead to community empowerment and long-term impact.^49^ Integrated interventions targeting schools or other non-residential sites, such as hospitals, religious sites or markets, require comprehensive planning and action. Such action needs appropriate combinations of interventions that are site-specific, effective, acceptable and affordable. Implementing integrated interventions requires collaboration among different sectors, capacity-building and leadership training of implementers to mobilize resources, form networks, and engage in participatory decision-making to ensure sustainability. 

In addition to the issues discussed above, some underlying factors (Fig. 1) need to be in place for integrated interventions to be effective, such as political will, funding, knowledge, capacity and empowered communities. Political will and funding largely depend on external factors, whereas facilitation of education and training and community engagement are technical aspects that can be adapted to the context of local communities. Community ownership of interventions to care for common environments and individual well-being must be based on bottom-up approaches, justified by social and behavioural theories.^50^ Fundamental factors in sustaining good integrated strategies would be strengthened by well-educated, confident, responsible and environmentally aware citizens who understand the holistic interrelationships between environment and disease and keeping neighbourhoods clean and healthy. On the other hand, physical accessibility and legal accountability criteria are important to select spaces that are suitable for integrated strategies where bottom-up community approaches or government-driven top-down approaches or both can be employed. Finally, and as a recommendation for future research, we have identified some gaps in knowledge that need to be addressed to strengthen the evidence base for best practices in management strategies for the integration of dengue and waterborne diarrhoeal diseases (Box 2).

Box 2 **Key knowledge gaps for integrated management of *Aedes*-borne arboviral diseases and waterborne diarrhoeal diseases**

- Do water, sanitation and hygiene initiatives and household water treatment and safe storage interventions benefit vector control?
- Would vector control interventions integrated into sanitation projects be effective?
- How effective is community waste disposal for integrated disease control?
- Could mosquito vectors that breed in polluted water and sewers be controlled through integrated sanitation projects?
- Can integrated disease management strategies offset potential disease risk increases due to climate change?
